# Chemical Composition and In Vitro Antioxidant and Antimicrobial Activities of the Marine Cyanolichen *Lichina pygmaea* Volatile Compounds

**DOI:** 10.3390/md20030169

**Published:** 2022-02-25

**Authors:** Hiba Sanad, Zahira Belattmania, Ahmed Nafis, Meryem Hassouani, Noureddine Mazoir, Abdeltif Reani, Lahcen Hassani, Vitor Vasconcelos, Brahim Sabour

**Affiliations:** 1 Laboratory of Plant Biotechnology, Ecology and Ecosystem Valorization—URL CNRST N°10, Faculty of Sciences El Jadida, University Chouaib Doukkali, P.O. Box 20, El Jadida 24000, Morocco; hiba.sanad.ucd@gmail.com (H.S.); belattmania.z@ucd.ac.ma (Z.B.); m.hassouani@gmail.com (M.H.); mazoirn@gmail.com (N.M.); abreani@yahoo.fr (A.R.); sabour.b@ucd.ac.ma (B.S.); 2 Department of Biology, Faculty of Sciences El Jadida, University Chouaib Doukkali, P.O. Box 20, El Jadida 24000, Morocco; ahmed.nafis@edu.uca.ac.ma; 3 CIIMAR, Interdisciplinary Centre of Marine and Environmental Research, University of Porto, Terminal de Cruzeiros do Porto de Leixões, Av. General Norton de Matos, s/n, 4450-208 Matosinhos, Portugal; 4 Laboratory of Microbial Biotechnologies, Agrosciences and Environment, Faculty of Sciences Semlalia, University Cadi Ayyad, P.O. Box 2390, Marrakech 40001, Morocco; lhassani@uca.ac.ma; 5 Department of Biology, Faculty of Sciences, University of Porto, Rua do Campo Alegre, 4169-007 Porto, Portugal

**Keywords:** volatile compounds, *Lichina pygmaea*, chemical composition, antioxidant and antimicrobial activities

## Abstract

Volatile compounds from the marine cyanolichen *Lichina pygmaea*, collected from the Moroccan Atlantic coast, were extracted by hydrodistillation and their putative chemical composition was investigated by gas chromatography coupled to mass spectrometry (GC/MS). Based on the obtained results, *Lichina pygmaea* volatile compounds (LPVCs) were mainly dominated by sesquiterpenes compounds, where *γ*-himachalene, *β*-himachalene, (*2E,4E*)-2,4 decadienal and *α*-himachalene were assumed to be the most abundant constituents, with percentage of 37.51%, 11.71%, 8.59% and 7.62%, respectively. LPVCs depicted significant antimicrobial activity against all tested strains (*Staphylococcus aureus* CCMM B3, *Pseudomonas aeruginosa* DSM 50090, *Escherichia coli* ATCC 8739 and *Candida albicans* CCMM-L4) with minimum inhibitory concentration (MIC) values within the range of 1.69–13.5 mg/mL. Moreover, this LPVC showed interesting scavenging effects on the 2,2-diphenyl-1-picrylhydrazyl radical with an IC_50_ of 0.21 mg/mL. LPVCs could be an approving resource with moderate antimicrobial potential and interesting antioxidant activity for cosmetics and pharmaceutical applications.

## 1. Introduction

Lichens, as symbiotic complex of autotrophic (microalgae, cyanobacteria) and heterotrophic (fungi), are highly adapted to extreme habitats comprising the coastal zones [1]. Indeed, marine lichens are exposed to an exceptionally range of pressures, due to daily tidal cycles that terrestrial lichens cannot resist [2]. The lichens growing on littoral and supralittoral zones differ in their resistance to the duration of exposure to salt water and are clearly distinguished taxonomically [1]. Some studies determined lichen zones without connecting them to tidal cycles [3], and others highlighted the significance of tidal periodicity [4,5,6]. Species of the genus *Lichina* are attractive marine lichens due to their outstanding habitat, commonly colonized by a relatively limited number of pyrenocarpous lichens (frequently crustose). *Lichina* is the only shrubby lichen and is associated with cyanobacterial photobionts, allowing both acquisition of inorganic carbon and nitrogen fixation [7]. The closely related marine *Lichina* species (*L. confinis* and *L. pygmaea*) have comparable distribution ranges in the Northeast Atlantic, usually co-occurring at the same rocky coast but inhabiting different littoral zones [8]. According to Smith, [9], *L. confinis* could reach higher latitudes (Norway and Iceland), whereas *L. pygmaea* could be found at lower latitudes (Morocco and Canary Islands).

**Figure 1:**
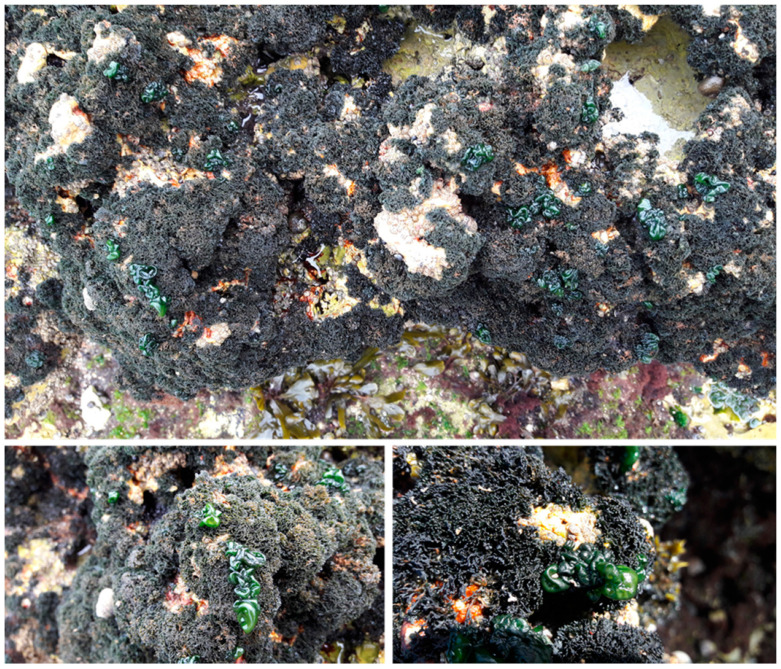
Photos of *Lichina pygmaea* epiphytized by flourished vesicles of its cyanobiont *Rivularia bullata*.

*L. pygmaea* (Lightf.) C. Agardh is a saxicolous fruticose lichen that occupies the upper intertidal zone, forming dark cushions some millimeters thick (Figure 1). It was historically considered to form a symbiosis with *Calothrix* [10], updated, afterwards, via molecular characterization to *Rivularia* [8]. *L. pygmaea* undergoes harmful UV radiation, exposure to sun and reverberation, salty conditions, immersion times, and wave actions. Consequently, this lichen could possess protective secondary metabolites with bioactive properties [11]. The majority of secondary metabolites in lichens are produced through the polyketide pathway and consist mainly of monocyclic phenols and bicyclic phenols attached by an ester bond [12]. Several lichens have been confirmed to be a source of important secondary metabolites for pharmaceutical applications [13]. There is growing interest in the pharmaceutical properties of compounds derived from lichens. However, fairly few lichen substances have been deeply monitored for biological activity and therapeutic potential; this is mainly due to the difficulties of having them in high quantities and adequate purities for structural elucidation and pharmacological tests [11]. It has been previously reported that lichen secondary metabolites exhibit antimicrobial, antioxidant, anti-inflammatory, cytotoxic, analgesic, antipyretic, and antiviral properties and could be potential sources of pharmaceutically useful chemicals [11]. In this context, the present study aims to evaluate, for the first time, the antioxidant and antimicrobial activities of volatile compounds of *Lichina pygmaea* harvested from the Moroccan Atlantic coast.

## 2. Results and Discussion

### 2.1. Chemical Composition of the Volatile Compounds

**Figure 2:**
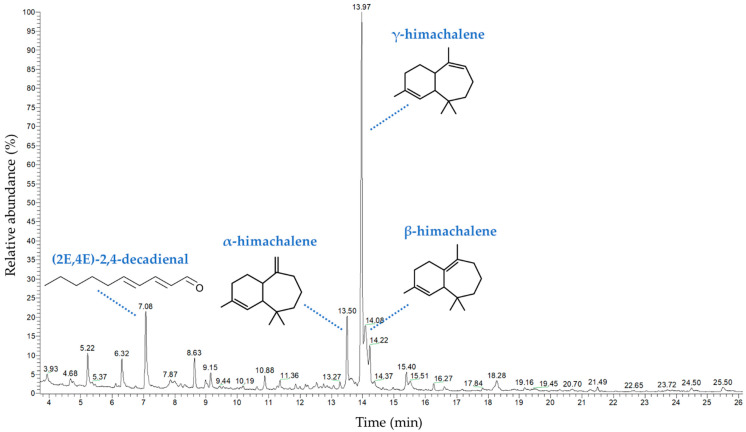
GC/MS chromatogram of *Lichina pygmaea* volatile compounds with putative chemical structures of the most abundant molecules.

The chemical components of *Lichina pygmaea* volatile compounds (LPVCs) were determined by GC/MS (Figure 2). In total, 25 volatile organic constituents were presumed (Table 1), which represent 93.54% of total volatile compounds (VCs). Sesquiterpenes, the major class of compounds, and *γ*-himachalene, *β*-himachalene, (*2E,4E*)-2,4-decadienal and α-himachalene seem to be the most abundant compounds, with percentages of 37.51%, 11.71%, 8.59% and 7.62%, respectively. Compared to the literature, LPVCs exhibit a special chemical composition in terms of volatile compounds. Kahriman et al. [14] showed that *Evernia prunastri* and *Evernia divaricata* EOs were characterized by monoterpene hydrocarbons (23.3 and 37.7%) and oxygenated monoterpenes (7 and 13.0%) as major constituents in the EO, respectively. Moreover, these compounds were *β*-pinene (6.3% and 8.0%), α-pinene (6.6%, 7.2%), limonene (1.6%, 6.3%), α-phellandrene (3.3%, 4.4%), respectively. Furthermore, Maqbul et al. [15] demonstrated that amino acid (38.1%) and cymene (29.1%) were the most volatile constituents in the lichen *Parmotrema perlatum* EO. The sesquiterpene (*E,E*)-2,4-decadienal present in LPVCs could be related to the associated cyanobiont, *Rivularia bullata*. This polyunsaturated aldehyde, usually reported in Diatoms microalgae [16], has also been identified in the Cyanobacteria *Anabaena* and *Microcystis* [17]. From the obtained results, it is obvious that VCs profile of *L. pygmaea* as marine lichen is significantly different from some previously investigated terrestrial lichens. The volatile organic compounds in marine macrophytes, released into the seawater, are implicated in the chemical communications process; these compounds have a vital role as pheromones or allelochemicals for communication and interaction with the surrounding environment [18,19]. The marine macrophytes species generate the volatile organic compounds depending to their physiology and ecosystem abiotic stresses [20].

### 2.2. Antioxidant Activity

**Figure 3:**
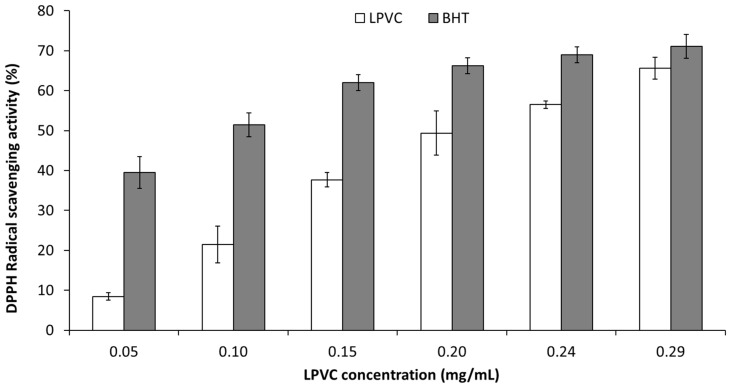
DPPH radical-scavenging activity of volatile compounds of *Lichina pygmaea* (LPVCs) compared to BHT.

The antioxidant potential of LPVCs compared to BHT was tested by DPPH radical scavenging assay (Figure 3). The LPVCs and BHT depicted radical scavenging inhibition of 65.58 and 71.12% at 0.29 mg/mL, respectively. The IC_50_ of the LPVCs (0.21 mg/mL) was relatively higher than that of the BHT (0.08 mg/mL). As shown in Figure 3, there was a positive correlation between DPPH radical scavenging activity and the concentration of the LPVC. Similar results have been reported for volatile compounds from some marine macrophytes [21]. Several studies were conducted on the antioxydant activities of *Lichina* extracts [22,23] but reports pointing to the antioxidant activity of its volatile compounds are lacking. The radical-scavenging ability is mainly related to the hydrogen atom providing capability of a compound and is not linked to the redox potentials alone [24]. The interesting antioxidant activity of LPVCs could be due to the presence of the sesquiterpene γ-himachalene and β-himachalene [25]. The minor components may also contribute to the activity of VC individually and/or synergistically [26,27]. Thus, the antioxidant activity of LPVCs could be attributed to the presence of α-longipinene and caryophyllene [28,29].

### 2.3. Antimicrobial Activity

*Lichina pygmaea* volatile compounds were screened for their antibacterial and antifungal activities using a diffusion disc method and a microdillution on microplates assay. Based on inhibition zones, the LPVCs showed potent antimicrobial activity towards all tested microorganisms, with hallo diameters ranging from 9.5 mm to 16 mm (Table 2). The highest zones were observed against *Candida albicans* (CCMM-L4) and *Staphylococcus aureus* (CCMM B3), with 16 mm and 14 mm, respectively. However, the lowest one was recorded for *Escherichia coli* (ATCC 8739), which was 9.5 mm as diameter. In addition, the antimicrobial efficiency of LPVCs was determined by measuring the minimum inhibitory concentration (MIC), as shown in Table 2. Particularly, a moderate antimicrobial activity was demonstrated against the Gram-negative bacteria *E. coli* with an MIC value of 1.69 mg/mL. The other tested strains exhibited similar MIC values of 13.5 mg/mL. Similarly, Maqbul et al. [15] reported that the clinical strains of *Escherichia* and *Pseudomonas* show promising susceptibility towards the *Parmotrema perlatum* EO. Previous studies suggested that the high resistance of Pseudomonas species was due to their capability to form biofilm, which is a physical barrier formed by exopolymeric substances [30]. In the literature, the Gram-negative bacteria were known for their resistance to the bioactive substances through the expression of certain enzymes inactivating antimicrobials or by non-enzymatic pathways such as the expression of genes responsible for efflux pumps and increased permeability or modifications of targets [31]. For the yeast *C. albicans*, their prolonged exposure to antifungals can promote the development of such tolerance and resistance by several mechanisms, including the formation of biofilms that decrease the accessibility of the antifungal, the selection of spontaneous mutations that increase the expression or decrease the sensitivity of the target and the ability to evade the host’s immune system [32]. Generally, volatile compounds from marine macrophytes act as chemical defenses against bacterial and fungal biofilms [33]. In another study, methanolic and ethyl acetate extracts from the lichen *Umbilicaria cylindrica* demonstrated important antimicrobial activity against eight strains, including *S. aureus*, *E. coli* and *C. albicans*, with MIC values ranging from 15.62 to 62.50 μg/mL [34]. Other reports indicated that extracts from the two lichens *Evernia prunastri* and *Pseudevernia furfuracea* exhibited a higher antibacterial effect vs. five clinical isolates of methicillin-resistant *S. aureus*, with MIC values of 0.039 to 0.15 mg/mL, and extracts of *Ramalina farinacea* possessed MIC values in the range of 0.078–0.625 mg/mL [35]. The antimicrobial effect of LPVC could be explained by the presence of a high level of the sesquiterpene himachalene derivatives, since a positive correlation between these compounds and the antimicrobial activity was proved [36]. These sesquiterpenes were very common in volatile compounds, especially those extracted from *Capsicum chinense*, *Cedrus atlantica* and *Cedrus libani* [37,38,39]. The antimicrobial activity could also be attributed to the presence of some lichen minor components. For example, caryophyllene oxide was known to possess interesting pharmacological properties such as antimicrobial and antioxidant activities [40,41,42]. Hence, we could confirm that LPVC is a potent weapon to fight multidrug-resistant and pathogenic strains.

## 3. Materials and Methods

### 3.1. Sampling and Volatile Compunds Extraction

*Lichina pygmaea* was collected in April 2021 at the upper midlittoral zone of the rocky shores of El Jadida (33°13′55.8″ N 8°33′24.8″ W), Atlantic coast of Morocco. The harvested lichen was washed with water and then drained for 24 h. The lichen biomass (100 g) was put into the glass container of hydrodistillation equipment, including 1 L of distilled water. The distillate was further mixed with dichloromethane solvent in a separating funnel, shaken vigorously for 20 min and then held until the two layers settled completely. After 2 h, the lower layer of the separating funnel with VC was collected and concentrated using a rotary evaporator. The extracted VC was dried using anhydrous sodium sulfate and stored in a closed glass vial at 4 °C for further use.

### 3.2. GC-MS Analysis

The quantitative and qualitative analysis of the chemical composition of LPVC was carried out using gas chromatography linked to mass spectrometry (GC-MS) (Trace 1300 Gas Chromatograph, Waltham, MA, USA), equipped with a Thermo Scientific TG-5MS capillary column (length 30 m; inner diameter 0.25 mm; film thickness 0.25 μm) and coupled to a mass selective detector (ISQ Single Quadrupole Mass spectrometer, ionization voltage 70 eV). He was used as carrier gas with a flow rate of 1 mL/min, and the injection volume was 1 μL (20 μL of VC diluted with 2 mL of hexane). The machine temperature was programmed as follows: 1 min at 100 °C, 100 to 260 °C at 4 °C/min and 10 min at 246 °C. The temperatures of the injector, transfer, source and quadrupole were 260 °C, 280 °C, 230 °C and 150 °C, respectively. The identification of volatile compounds was carried out by comparing their mass spectra with the reference spectra contained in the NIST database and the Adams terpenes library [43]. In addition, their retention indices (RI) were determined in relation to a series of alkanes (C9 to C24) [44].

### 3.3. Antioxidant Activity

The antioxidant activity of LPVC was evaluated as 2,2-diphenyl-1-picrylhydrazyl (DPPH) free-radical scavenging according to the method slightly modified from Blois et al., [45]. Methanolic DPPH solution (0.06 mM) was added to LPVC at different concentrations. The samples were kept for 30 min in the dark at room temperature. Thereafter, the absorbance was measured at 517 nm using a spectrophotometer (UV-Visible Metashe 5200 HPC). The results were compared to a positive control (BHT). The percentage of DPPH radical scavenging was calculated with the following equation:

DPPH scavenging activity (%) = [(Ac − As)/Ac] × 100

where Ac is the absorbance of the negative control (DPPH methanolic solution), and As is the absorbance of the sample.

### 3.4. Antimicrobial Activity

In order to assess the antimicrobial activity of LPVCs, three strains of resistant bacteria and a single strain of pathogenic yeast were used. The bacterial strains are named *Staphylococcus aureus* (CCMM B3), *Pseudomonas aeruginosa* (DSM 50090) and *Escherichia coli* (ATCC 8739). *Candida albicans* CCMM-L4 of vaginal origin was the only pathogenic yeast [46] used in this study. To qualitatively determine the antimicrobial activity of *Lichina pygmaea* VC, the disc diffusion method was used, as described previously by Nafis et al. [47]. From a fresh culture of 24–48 h on liquid Mueller–Hinton (MH) for bacteria and Sabouraud (SD) for yeast, an inoculum of bacteria and yeast was prepared in sterile physiological water (9‰ NaCl). Petri dishes containing the two culture media were inoculated using the already prepared inoculum. The excess was removed, and the Petri dishes were then dried. Sterile discs of 6 mm diameter were impregnated with 20 μL of LPVC and placed on the surface of the agar already inoculated. Then, the Petri dishes were placed at 4 °C for 2 h to stop the growth of the test microorganisms and promote the diffusion of secondary metabolites of LPVC. The Petri dishes were incubated at 37 °C for 24 h for bacteria and at 28 °C for 48 h for yeasts. After incubation, a zone or a clear halo is present around the discs if the microbial growth is inhibited by *Lichina pygmaea* VC. Ciprofloxacin and fluconazol were used as positive controls. This test was performed in three repetitions. To quantitatively evaluate the antimicrobial activity of LPVC, the microdilution method was used [48]. From an overnight culture of each microorganism, microbial suspensions were prepared with the required concentration of 106 CFU /mL for bacteria and (1–2 10^3^ cells/mL) for yeast. Then, the dilution of 1/50th was carried out by adding 100 μL of the microbial suspension in 4900 μL of the culture medium suitable for each test microorganism. Serial ½ dilutions (from 52 mg/mL to 0.21 mg/mL) of LPVC were prepared in sterile test tubes containing Mueller–Hinton broth (MHB) for bacteria and Sabouraud broth (SDB) for yeast and Dimethylsulfoxide (DMSO) at 2%. Then, 96-well plates were prepared by distributing in each well 100 μL of the 1/50th microbial suspension and 100 μL of each dilution of LPVC. The plates were then incubated at the optimum temperature for growth of the test microorganism and for the time suitable for its growth. MIC, the lowest concentration of LPVC, was the equivalent of the well that did not contain any visible microbial growth (absence of turbidity).

## 4. Conclusions

The present study showed that *Lichina pygmaea* volatile compounds exhibit high levels of sesquiterpenes compounds with significant antioxidant activities and moderate antimicrobial potential. The presence of himachalene derivatives could enhance the antibacterial activity, whereas the antioxidant and scavenging effects of LPVC could be related to some other substances such as α-longipinene and caryophyllene. These interesting properties of LPVC can make it a potential candidate for pharmaceutical and cosmetic uses.

## Figures and Tables

**Table 1:** Chemical composition of volatile constituents extracted from *Lichina pygmaea*.

RI	Coumpound Name	Relative Abundance (%)
769	2,3-dibutyloxirane	1.14
1258	1-decanol	0.76
1250	(*Z*)-2-decenal	3.13
1261	(*E*)-2-decenal	0.3
1311	(*E,E*)-2,4-decadienal	4.1
1314	**(*2E,4E*)-2,4-decadienal**	**8.59**
1315	(*E*)-2-pentenol	0.35
1350	limonene oxide	0.43
1364	2-undecenal	2.71
1380	(*E*)-4,5-epoxy-2-decenal	2.3
1469	*α*-longipinene	0.31
1473	*ς*-muurolene	0.31
1478	*E-β*-farnesene	1.45
1484	1-methyl-4-(6-methylheptan-2-yl)benzene	0.93
1490	4,5-di-epi-aristolochene	0.36
1506	(*R*)-cuparene	0.61
1663	***α*** **-himachalene**	**7.62**
1708	***γ*** **-himachalene**	**37.51**
1723	***β*** **-himachalene**	**11.71**
1730	1,3,5-himachalatriene	3.25
1735	naphthalene	1.77
1770	guaiazulene	1.26
1986	caryophyllene oxide	0.35
2233	cadalene	1.72
2913	n-hexadecanoic acid	0.57
	Total	93.54

RI: Retention index measured relative to n-alkanes (C-9 to C-24) on a non-polar TG-5MS column.

**Table 2:** Inhibition zone diameters and MIC of *Lichina pygmaea* volatile compounds.

	*Lichina pygmaea* Volatile Compounds		MIC Ciprofloxacine	MIC Fluconazol
	IZ	MIC		
Gram positive bacteria				
*S. aureus*(CCMM B3)	14.0 ± 0.33	13.5 ± 0.00	0.01 ± 0.00	-
Gram negative bacteria				
*E. coli* (ATCC 8739)	9.5 ± 0.26	1.69 ± 0.00	0.06 ± 0.00	-
*P. aeruginosa* (DSM 50090)	11.0 ± 0.78	13.5 ± 0.00	0.25 ± 0.00	-
Pathognic yeast				
*C. albicans* (CCMM-L4)	16.0 ± 0.24	13.5 ± 0.00	-	1 ± 0.00

IZ: Diameter of inhibition zone including disc diameter of 6 mm, by the agar disc diffusion method at a concentration of 20 μL of VC/disc and a concentration of 10 μg/disc and 5 μg/disc for fluconazol and ciprofloxacin, respectively. MIC: minimum inhibitory concentration in mg/mL.
